# The Application Value of Personalized 3D Printed Navigation Molds in Neurosurgery

**DOI:** 10.1055/a-2564-1309

**Published:** 2026-02-12

**Authors:** Lijie Hou, Jian Zhang, Ke Meng, Yunpeng Xue, Xin Feng

**Affiliations:** 1 School of Life Science and Technology, Changchun University of Science and Technology, ChangChun City, China; 2 Department of Neurosurgery, The Third Bethune Hospital of JiLin University, ChangChun City, China

**Keywords:** 3D printed navigation molds, neurosurgery, precise positioning, intracerebral hematoma, hydrocephalus, brain abscess

## Abstract

**Background:**

Many neurosurgical procedures are expensive and technically demanding. The incidence of complications is high. Based on the advantages of three-dimensional (3D) technology in surgical procedures, it is necessary to develop personalized navigation molds that use 3D printing technology and to analyze the clinical application of this technology in intracerebral hemorrhage, hydrocephalus, and brain abscess.

**Methods:**

According to the patient-specific brain computed tomography imaging data, we reconstructed intracranial lesions or target structures and important brain regions. By this, neurosurgeons can accurately locate the intracranial lesions under conditions equivalent to direct vision, analyze the morphology and size of lesions, and perform precise lesion puncture. Based on patient-specific differences, we designed the best 3D printed navigation molds to improve the accuracy of intracranial lesion targeting, analyzed the sources of deviation, and provided clinical application basis.

**Results:**

Five cases of intracerebral hematoma, two cases of hydrocephalus, and two cases of brain abscess were treated between September 2020 to January 2022. Lesion puncture and drainage were performed with 3D printed navigation molds. The average puncture error was 2.86 mm. All nine patients recovered well after surgery, and no infection or death occurred.

**Conclusion:**

The personalized 3D printed navigation molds used in this study are beneficial to accurately locate intracranial lesions or target 3D structures during surgery.

## Introduction

Ventriculoperitoneal shunt is an effective method for the treatment of hydrocephalus. However, if the location or path of the ventricular catheter is not good, postoperative complications can occur: Improper positioning of catheter too close to the choroid plexus or against the ventricular wall is a common cause of shunt obstruction. 1 The typical location of primary intracerebral hematoma is in the basal ganglia area, and the hematoma has different shapes and sizes. 2 The STICH (Surgical Treatment for Cerebral Hemorrhage) I and II studies had shown that craniotomy did not improve neurological outcomes or reduce mortality if compared with conservative management. 3 In other studies, it has been shown that reducing the initial hematoma volume by at least 70% to <15 mL by hematoma puncture and thrombolysis might improve functional outcome. 4 5 Some studies have shown that puncture and aspiration of brain abscesses is as effective and less invasive than craniotomy. 6 Therefore, minimally invasive abscess puncture has become the preferred surgical method.

The key to the success of minimally invasive techniques in these lesions lies in the accuracy of the puncture. Neuronavigation, framebased stereotaxy, computed tomography (CT) imaging data surface positioning and robotic assistance help to increase the accuracy. 7 8 9 10 These techniques are expensive and difficult to popularize in primary and secondary care hospitals. Therefore, a method with relatively simple technology, low price, and high accuracy is needed.

Studies have shown that 3D printing technology is helpful in surgical procedures with good clinical outcomes. Although some previous studies have shown that minimally invasive puncture surgery can effectively treat cerebral hemorrhage, hydrocephalus, and brain abscess, there is currently no research on the combination of minimally invasive puncture and 3D technology with focus on accuracy, convenience, and costs. 10 11 Therefore, the current study was initiated.

## Materials and Methods

### Patients

This study included nine patients who underwent 3D-printed navigation mold-assisted puncture and drainage at the Neurosurgery Department of The Third Bethune Hospital of JiLin University between September 2020 and January 2022. Among them, five patients had intracerebral hematoma, two patients had brain abscess, and two patients with hydrocephalus. The surgery was approved by the patient's family. and the study was approved by the Ethics Committee of Jilin University Sino-Japanese Friendship Hospital (also known as The Third Bethune Hospital of JiLin University), and all patients or their families gave informed consent. The detailed clinical information of patients is showed in Table 1 .

**Table 1:** Clinical data

Case no.	Gender	Age (yr)	Diagnosis	Coma	Preoperative lesion volume (mL)
1	M	33	Intracerebral hematoma	No	26.5
2	M	61	Intracerebral hematoma	No	39.8
3	M	53	Intracerebral hematoma	No	32.89
4	M	59	Intracerebral hematoma	Yes	36.54
5	F	80	Intracerebral hematoma	No	27.37
6	F	59	Hydrocephalus	No	–
7	F	16	Hydrocephalus	No	–
8	M	61	Brain abscess	No	13.85
9	F	34	Brain abscess	No	38.76

Abbreviations: F, female; M, male.

### Construction of Personalized 3D Printed Navigation Molds

The 3D printer (A8S model) was purchased from China GuangDong ShenZhen Aurora Innovation Technology Co., LTD (Changchun, China). The printing consumables were solid polylactic acid (PLA) material.

#### 3D Reconstruction of Intracranial Lesions

Thin-slice CT was performed in all patients. The images were stored in DICOM (Digital Imaging and Communications in Medicine) files and imported into 3D Slicer software. The patient's skull, skin, intracranial lesions, and target structures were segmented according to the original proportions, differentiated by different colors, and then reconstructed in three dimensions.

#### Design of 3D Printed Navigation Molds

The puncture target was determined by defining the entry point and the trajectory to the lesion center. Important blood vessels and functional areas were avoided. The guide tube diameter of the navigation molds was larger than the diameter of the catheter for puncture and drainage. The navigation molds were designed according to the angle of the puncture path and the patient's personalized craniofacial features.

#### Acquisition of 3D Printed Navigation Molds

Using the reconstructed skin as the inner surface, the personalized navigation molds with a thickness of 3 mm were acquired, with the mask closely fitting to the patient's face, ensuring the accuracy of puncture. After laterally sectioning one-half of the navigation mold puncture channel (which then matches the drainage catheter), the acquisition was stopped when the navigation molds contained maxillofacial positioning structures such as orbit and nose root, etc., for fixation. This aim was to save printing time, and the puncture depth was determined by the measured distance in the software.

#### Printing of 3D Navigation Molds

The designed navigation molds were exported as STL file and then exported as GCODE file after setting printing parameters in the slicing software, and then 3D printing was performed. The printing equipment met the following conditions: (1) layer thickness ≤ 0.3 mm; (2) printing accuracy ≤ 0.1 mm; (3) printing error (deformation rate, 3D offset) ≤ 5%. Moreover, the guide plate should be perfectly matched and attached to the surgical site. After installation, there should be no warping or loosening, and the surface should be smooth without any residual support materials or powder debris.

The size of the navigation molds affected the printing time. Without loss of accuracy, the size of the mold could be adjusted to only retain some clear and unmovable positioning marks, while closely fitting the face. In doing so, the printing area is minimized and the preoperative preparation time is shortened.

#### Disinfection of 3D Printed Navigation Molds

To effectively prevent pollution and infection, the designed navigation molds must be disinfected before use. The PLA material selected for the navigation molds had the characteristics of being resistant to high temperature and humidity. In addition, they were disinfected or sterilized using low-temperature plasma and ethylene oxide disinfection method to ensure the sterile operation of the surgery.

### Lesion Puncture and Drainage with Assistance of 3D Printed Navigation Molds

The patients were placed in a supine position. After induction of general anesthesia, the operative field was disinfected. The disinfected navigation mold was covered with sterile film and attached to the patient's craniofacial positioning structure. The operation was performed according to the trajectory and planned depth of the guidance tube of the navigation mold. Thin-slice CT scan of the head was performed after surgery to evaluate the efficacy of the operation.

### Lesion Clearance Rate and Puncture Error

Preoperative and postoperative thin-slice CT data of the head were imported into the 3D Slicer, and Volume Rendering was used for 3D reconstruction to obtain the volume of the lesion or target structures 12 and 3D images of the implanted catheters. The clearance rate was calculated according to the changes in the volume of the lesion before and after surgery. The tip of the catheter was taken as the standard. After calibration by Line tool in 3D Slicer software, the distance between the catheter tip and position in 3D design was obtained. The distance between the actual catheter tip and the preplanned target was the puncture error, and the average value was taken. The smaller the puncture error, the higher the positioning accuracy.

## Results

### Surgical Outcomes

All nine surgeries were successfully completed, and postoperative CT showed the catheter tip in the lesion. The average puncture error was 2.86 mm ( Table 2 ),. and met the standard puncture accuracy need. 13 At the same time, the average clearance rate was > 70%. The reason for large puncture error in patients with brain abscess was the displacement of catheter tip when entering the capsule of the abscess. The reason for the low brain abscess clearance rate was considered to be the rigidity of the abscess capsule and smaller volumes of aspiration. The deviation of the ventricular catheter in shunt surgery was more likely to be caused by the drift in the cerebrospinal fluid (CSF), but it conformed to the predetermined target.

**Table 2:** Lesion clearance rate and accuracy

Case no.	Preoperative lesion volume (mL)	Postoperative lesion volume (mL)	Lesion clearance rate (%)	Deviation distance (mm)
1	26.5	6.62	75	1.43
2	39.8	11.16	72	3.17
3	32.89	9.13	72	4.65
4	36.54	8.77	76	1.70
5	27.37	7.65	72	2.46
6	–	–	–	3.36
7	–	–	–	1.55
8	13.85	7.65	45	3.58
9	38.76	7.23	81	3.85

### Representative Cases

#### Intracerebral Hemorrhage

**Fig. 1:**
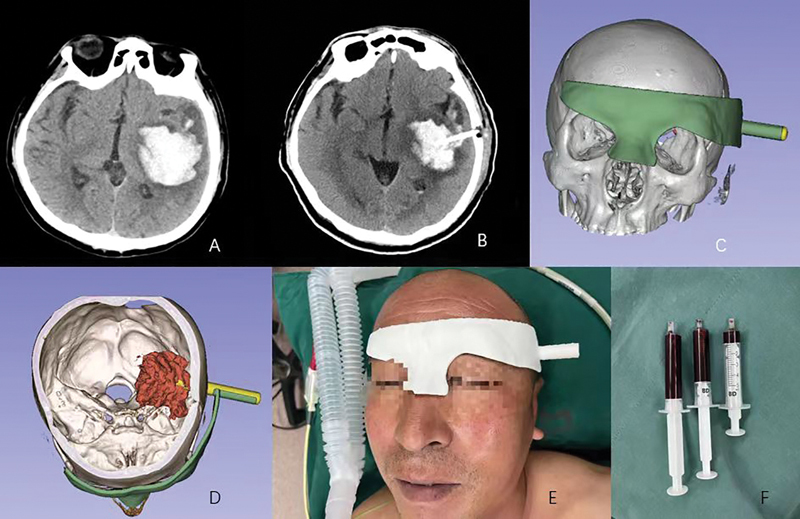
Patient with intracerebral hemorrhage. ( **A** ) Preoperative computed tomography (CT); ( **B** ) postoperative CT; ( **C** – **D** ) planning of 3D printed navigation molds and reconstruction; ( **E** ) 3D printed navigation mold and surgical guidance of craniofacial area and hematoma; ( **F** ) about 11-mL hematoma was aspirated after catheter puncture.

The patient had an intracerebral hemorrhage ( Fig. 1 ). After excluding an underlying vascular lesion by CT angiography, a transtemporal approach was planned according to the morphology and location of the hematoma. Before surgery, we reconstructed the patient's skull (gray part), hematoma (red part) and planned 3D-printed navigation mold (green part). In addition, the surgical path was planned (yellow part, Fig. 1C, D ). The navigation mold was placed over the head to accurately locate surgical target site of the patient ( Fig. 1E ). After the catheter was placed, 11 mL of hematoma was smoothly aspirated ( Fig. 1F ). The postoperative CT showed a good placement of the catheter ( Fig. 1B ).

#### Hydrocephalus

**Fig. 2:**
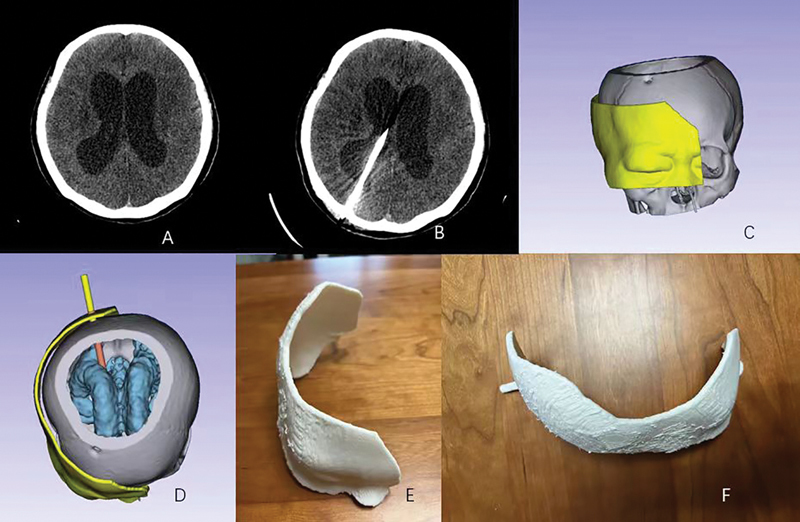
Patient with hydrocephalus. ( **A** ) Preoperative computed tomography (CT); ( **B** ) postoperative CT; ( **C** – **D** ) definition of the angle between occiput and occipital horn and planning of navigation mold; ( **E** – **F** ) 3D printed navigation mold.

The patient had a hydrocephalus ( Fig. 2 ). Preoperative CT ( Fig. 2A ) showed obvious dilatation of both lateral ventricles. Before surgery, the skull (gray part), ventricular system (blue part), and planned navigation mold (yellow part) containing nasal roots eyebrow arches, and tuber parietale were reconstructed ( Fig. 2C, D ). The surgical path is shown in red ( Fig. 2D ). The navigation mold was 3D printed ( Fig. 2E, F ). The postoperative CT showed a good placement of the ventricular catheter ( Fig. 2B ).

#### Brain Abscess

**Fig. 3:**
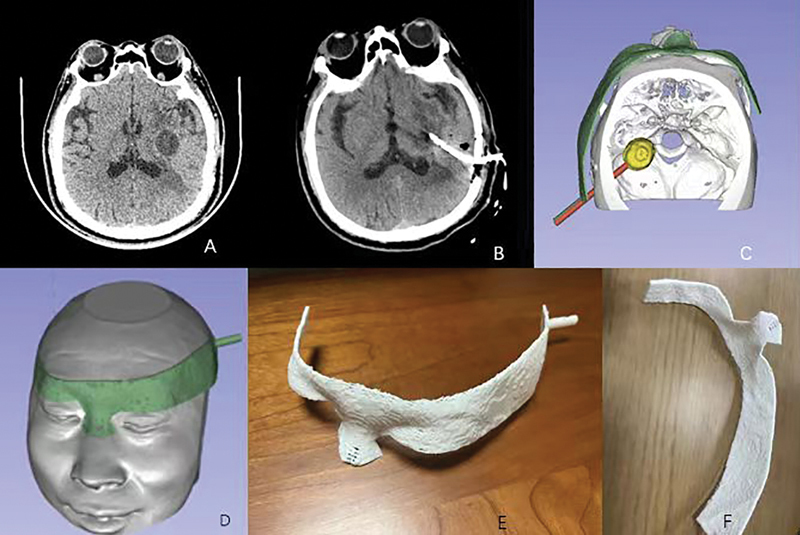
Patient with brain abscess. ( **A** ) preoperative computed tomography (CT); ( **B** ) postoperative CT; ( **C** ) definition of the trajectory to puncture the abscess; the volume rendering module in 3D Slicer was used, which aims to see the puncture path and the reconstructed abscess from above; ( **D** ) navigation mold planned; ( **E** ) 3D printed navigation mold; ( **F** ) Inside view of the 3D printed navigation mold; while ensuring the stability of the mold, a 0.3-mm precision printing was used to save time. The supporting materials were all set on the outer surface of the mold, and the inner surface inside was smooth, causing no damage to the patient's skin.

The patient had a brain abscess ( Fig. 3 ). Preoperative CT showed a circular low-density lesion in the left basal ganglia ( Fig. 3A ). Before surgery, the brain abscess (yellow part, Fig. 3C ), the surgical path (red part, Fig. 3C ), and the navigation mold were reconstructed (green part, Fig. 3D ). The navigational mold was applied during the operation ( Fig. 3E, F ). Postoperative CT showed a good position of catheter ( Fig. 3B ).

## Discussion

With the advancement of technology, cutting-edge tools such as specialized anatomical models and clinical applications are increasingly being used in the clinical work of neurosurgery. 14 Ventriculoperitoneal CSF shunting is a common procedure in hydrocephalus, including normal pressure hydrocephalus. 15 16 Shunt tube obstruction is a common complication. A recent study showed that a common cause of dysfunction is catheter ostruction by debris (occurring in the fist month after surgery) and by choroidal plexus (occurring within 3 to 6 months after surgery), which might be related to improper placement of the catheter. 17 18 Using 3D printing navigation technology, a mold with guide holes can be designed based on the patient's facial anatomy. By accurately defining the trajectory, improper catheter placement close to the choroid plexus and repeated puncture attempts with the higher risk of debris can be prevented. 19

Hypertensive intracerebral hemorrhage are treated either by craniotomy or minimally invasive surgery. 20 21 Due to the different hematoma localizations, it is necessary to define the best surgical path.

Brain abscesses are treated by puncture and drainage. 11 Stereotactic puncture and drainage can identify pathogens and reduce the size of abscess. Postoperative bleeding is a risk of abscess puncture and drainage because of lacking visual control during operation. 22 23 By using 3D modeling software and the imaging data, precise analysis of each patient's lesion can be achieved. Using 3D navigation molds, personalized trajectories can be planned. Due to the high accuracy of the method, initially planned trajectories can be reused in case of abscess recurrence alleviating the need for a new burr hole.

The application of 3D printing navigation technology in medicine is increasingly integrated, and this technology has great potential due to its flexibility, diversity, and creativity. Based on the research of Ballard et al, 24 3D-printed anatomical models and surgical guides have been shown to reduce surgical time and operating room costs. In this research work, full consideration was given to the issue of the medical environment in primary and secondary care hospitals with its deficiencies in equipment, knowledge and routine in translating 2D CT data into 3D anatomy.. 9 With the use of the low-cost 3D printing navigation technology, accuracy of the surgical procedure can be increased and time can be saved, For the printing of the 3D printing navigation molds, we choose PLA powder. PLA powder has a good biodegradability and allows to form precise geometric shapes required for thermoforming and compression molds. The printing process with PLA powder is safe and nontoxic. In addition, the melting temperature of PLA powder is lower, which helps to reduce energy consumption and costs. The cost of printing are approximately 50 yuan (7.01 USD). 25 In recent years, the use of 3D printing navigation technology for minimally invasive catheter placement in the treatment of cerebral hemorrhage had been reported. 26 In this study, nine patients with different diseases were successfully treated. The 3D printing navigation molds were used to exactly guide the placement of catheters into hematoma, abscess or ventricular system. 3D printing navigation molds are a cheap alternative to neuronavigation and robot assistance. However, the technology also faces challenges. Due to the increased demand for this technology, neurosurgeons must not only be skilled users of modeling-related software, but also be able to perform the modeling and printing work quickly.

In this paper, we used the 3D printing navigation technology in the treatment of hydrocephalus, hematoma and brain abscess. Further applications in neurosurgery, such as balloon compression of trigeminal neuralgia and the treatment of various tumors, seem possible. 27 28 In addition, the combination of neuroendoscopes with other devices can further broaden the scope of application of 3D printing navigation technology in neurosurgery.

This study is a single-center retrospective study. The results may be biased due to the small sample size. It is recommended to validate the technology in a higher number of centers. 3D printing navigation technology enables surgeons to achieve highly personalized treatment while reducing technical requirements. However, it requires doctors to additionally learn 3D modeling and data application-related technologies.

## Conclusions

In this paper, 3D printing navigation molds designed based on the individual patient anatomy were used as an auxiliary surgical instrument for minimally invasive puncture of intracerebral hematoma, dilated ventricular system and brain abscess. The preoperative visual simulation in 3D and the navigation molds can help neurosurgeons to plan the optimal trajectory of catheter puncture.
